# Exposed Pulps

**Published:** 1866-11

**Authors:** Henry S. Chase


### EXPOSED PULPS.

BY HENRY S. CHASE, D. D. S., M. D.

Read before the Iowa State Dental Association.

So much has been said upon this subject recently that some may consider its discussion uncalled for at this time; yet we must continue to talk upon familiar topics, for those are they which most concern us, and for the better understanding of which we must continue to investigate.

Teeth with exposed pulps should seldom be extracted. A few years since extraction was the common mode of curing tooth-ache. Now we hope it is daily growing less frequent. Just as rapidly as dentists understand and practice the true principles of therapeutics, just so fast will people desire and demand of the profession the salvation of their natural teeth.

The diagnosis is not obscure; there is pain of a peculiar character, sharp, plunging, and nocturnal. The patient tells you "the nerve is bare." Pain is aggravated by contact of solids or liquids, or even the air itself. There is inflammation of the pulp, and swelling, it forces itself through the smaller orifice communicating with the cavity of decay, becomes strangulated, and pain of the most aggravated character ensues. Suppuration from the surface sometimes occurs, and you will see pus in the cavity of decay. Sloughing of the part frequently takes place, and then the pain ceases for the present, only to be again renewed on the first exciting cause. These "fits of tooth-ache" may occur every few weeks, until the inflamatory process and sloughing entirely destroy the pulp and its appendages.

I have said that the diagnosis is not obscure. But one will occasionally fail if not careful.

CASE.

Mr.—— refers to the right upper first and second molars as the seat of tooth-ache. Pain aggravated by cold and water, or drawing over them cold air. These teeth are in close contact from grinding surface to neck. One is plugged on its anterior proximal surface, and the other on its posterior. As plugging did not relieve the pain, the patient returned to his dentist. It was then discovered that these teeth had exposed pulps through cavities on their surfaces of mutual contact.

TREATMENT.—This may be comprised under the following heads:

Capping the exposed portion.
Excising the exposed portion.
Cauterization of the Pulp.
Cauterization and removal of the Pulp.
Cauterization and removal of the Pulp and its ramifications.

Capping was formerly resorted to by a majority of the profession who attempted any operation but extraction; and is still continued to some extent.

My personal experience in it has been limited, and is unfavorable to the operation.

The results of the practice of others which I have seen, has also confirmed me in the belief of its worthlessness. I have rarely known a living pulp treated in this way that was not often inflamed afterwards. Those teeth that were comfortable had lost their pulp vitality. And yet many that had dead pulps were uncomfortable, having degenerated into the low vitality of alveolar abscess.

Under the most favorable circumstances, capping is of doubtful utility. The only case where it is excusable is in that of a freshly exposed pulp, produced by excavating a cavity of decay. If no blood is drawn there is some chance of success. Place over the exposed pulp, and in *close contact with the opening*, a thin slice of Hill's stopping, lead, tin, or other non-conducting substance, a *little* moistened with creosote, carbolic acid, or tannin. The non conductor, however, should not be of perishable material. It is often recommended and practiced, to *arch* over the exposed pulp. I said a little while since, that inflamed pulps swell and crowd through the opening into the cavity of decay, become strangulated, and die. Therefore, it is much better to cap, so as to keep the pulp in its own home.

In all cases of pulp-treatment a temporary plug should be used, for a longer or shorter time, before plugging permanently.

EXCISION.—This is a new mode introduced, I think, by Dr. Allport, of Chicago. I believe it consists in scooping out a convex-shaped bit of the exposed pulp, thereby leaving a concavity in that which remains. This bleeds and heals by granulation, and is then capped. Judging by its analogue in general surgery, I should suppose that the concave portion would fill up to its original size. If so, that peculiarity of the operation would be useless. What success has attended this practice I am unable to say. If it should prove worthy of imitation, doubtless the process and its results will be made public through the Dental Journals.

CAUTERIZATION.—I hope this practice is discarded even by *laggards* in our profession. "Killing the nerve," and leaving it in the tooth to decompose under a recently introduced plug was at one time very common. At the commencement of my practice, twenty three years ago, it was in vogue. It was expected that two thirds of those thus treated would have alveolar abscess. But as alveolar abscess was sometimes cured, either spontaneously or by art, the risk was often taken by the patient willingly.

CAUTERIZATION and REMOVAL of the Pulp and its RAMIFICATIONS. This is the most successful and popular method of the day. Many young dentists suppose this to be a *very modern* operation; but by looking over the older dental journals they will see that it was, to some extent, practiced twenty years ago. I remember destroying and removing the pulp and root-vessels, of a right upper first bicuspid, for a lady in 1843. I plugged it with gold at the time. A year ago the tooth needed refilling, having a corner broken by mastication, and it is now in good working order.

It is only within a few years, however, that this operation has become common. It would have been so long ago, had the same fraternal feelings existed in the profession then as now. But dentists in those days were more disposed to keep "a good thing" to themselves, than to communicate it to their brethren.

The per cent. of teeth lost by this treatment is small indeed; some have greater success than others, owing to slight differences in *detail*, but all have such results as to warrant them in attempting to save nearly every tooth presented for treatment.

As an escharotic or devitalizer, arsenic is generally used. Some prefer cobalt; but, as the latter is used in a crude state, its escharotic properties are undoubtedly due to the arsenic with which it is always found in combination in the native ore. I have never employed cobalt, being quite satisfied with the action of pure arsenic. This, triturated with creosote or carbolic acid, together with a little sulphate of tannin, and sulphate of morphia, is the preparation I use. I do not know that the tannin and morphia do any good, but think they may lessen the pain of death.

Formerly, some killed and extirpated with an instrument. Some pretend to now. I pity their patients.

Arsenic, finely divided in creosote or carbolic acid, easily penetrates a very small opening to the pulp, and speedy action ensues. Very little preparation of the cavity is necessary. Removal of food, by an excavator, or warm water from a syringe, is sufficient.

After introducing the escharotic upon a bit of lint the size of a pin head, plug the cavity with a pellet of cotton dipped in sandarac varnish. This will hold the preparation safely, and is much better than wax. A pellet of cotton, thus saturated with sandarac, hardens *under water*, and makes a very good temporary plug for a few days, and even weeks.

Care should be exercised in introducing the arsenic, so as not to get too much, which might thus be forced out on the surrounding tissues. I have known severe peridentitis result from arsenic thus forced up between the gum and tooth.

My practice is to remove the escharotic at the end of twenty-four hours, and also the pulp, if dead; if it is not entirely destroyed, I remove that which can be without pain, and apply arsenic again.

By mistake or negligence of patients, I have had cases where the arsenic was retained one, two, three, or four weeks without apparent injury.

Greater mischief is likely to result from the retention of the dead pulp, than from that of the arsenic.

Arsenic probably rarely acts for a greater distance than the foramina of the roots, and seldom will its first application devitalize more than the pulp contained in the cavity itself.

To destroy the nerves and vessels of the roots, I find a second application necessary, after the removal of the pulp from its cavity.

The arsenious acid has such an affinity for the albumen of the pulp that it unites with it to *satisfaction*, forming an arsenite of albumen. If the pulp is swollen so as to *fill* its cavity, thereby preventing the action of the arsenic only upon the exposed portion of the pulp, the probability is that only that portion will be destroyed. On the other hand, if the pulp is *not* swollen, and there is room for the arsenical preparation to pass through into the pulp cavity, the greater surface which the pulp will then present to the action of the escharotic will insure its speedy death.

I think this accounts for the tardy and imperfect devitalization of the pulp in some cases. It is pretty generally known that the pulp is more effectually destroyed by arsenic, when not inflamed at the time of its application.

In other cases may not the limited action of arsenic be accounted for in this way, namely: the arsenic finds a superabundance of albumen in the pulp with which it is in contact and is easily satisfied; its corrosive action ceases and cannot be renewed until the dead portion of the pulp is removed either by an instrument or by a natural process of sloughing, when a clean surface being presented again to the contact of arsenic, a similar result is again produced. In this way, little by little, we sometimes destroy the pulp. A tedious operation.

When the pulp proper behaves in this way, the root-vessels are likely to do the same, and one is likely to give up the case in that respect. Here is where fine broaches must be used for extracting the living root-vessels, if we are determined to remove them.

The pain produced by destroying the pulp with arsenic does not exceed three hours; sometimes it lasts only half an hour. In only one-third of the cases is any pain produced.

The general impression is that not only the pulp proper, but also its appendages in the root canals should be extirpated before plugging.

When this is done the root canals are plugged as well as the pulp cavity. Some plug the canals with cotton or lint moistened with creosote; others use gold, tin, or Hill's stopping. Some medicate the pulp cavity with creosote, tannin, or iodine, for a few days before. Others plug immediately. Now it is certain that no one can say, with certainty, that this or that is the best method, because we have not the *recorded* experience of different operators to compare and draw our conclusions from.

If those who keep a true and minute record of the treatment of exposed pulps would give those results to the profession through the dental journals, we could learn something of value. Each one will travel his own road until he thinks he has found a better one. Here is mine.

CAUTERIZATION and REMOVAL of the PULP. I do not attempt to destroy the contents of the roots, or remove them. I prefer to let them live.

It is an *easy* practice.

It is a successful practice.

I only speak from my *own* experience. *Theoretically*, I believe in retaining *all* the pulp alive. Practically, I believe in retaining all the pulp and vessels alive which experience will warrant.

My practice is to destroy only the pulp proper, with one or two applications of the arsenical paste. Then remove all the pulp in the cavity, and as much of the canal contents as are dead. Syringe with warm and cold water; fill the cavity with tincture of the sulphate of tannin, and plug with cotton and sandarac varnish for one week. At the expiration of the time plug permanently, if a cheap metal is to be used; if gold is to be the permanent plug, I plug temporarily with tin foil, or Hill's stopping.

If the remaining root vessels are sensitive to cold water, defer plugging until the sensitiveness is cured.

Do the root vessels die after plugging? I think not; as I have found them alive after three months' trial with a temporary plug.

Does alveolar abscess occur after plugging? In some cases, but 1 think not oftener than where the vessels are extirpated.

Out of seventy cases treated in this manner only two suppurated. One was in consequence of peridentitis following immediately an illness of acute rheumatism lasting two months.

There can be no doubt that the peridentitis was a legitimate result of rheumatism. I did not see the patient until suppuration had taken place. The other case was the suppuration of an under molar.

In both cases the teeth are retained without further discomfort, although the suppuration occurred some months since.

In two other cases there was slight peridentitis, which yielded promptly to mercury.

No case of *extraction* has occurred as a result of pulp-treatment in the last one hundred cases.

Excuse me for seeming egotistical; but I must say that there is *no guess work* in making up the results of my practice. *I record every case*, and keep my record in the following manner.

Take a blank book and rule two open opposite pages for the following heads, each of which, write perpendicularly over its own column, making the columns of a width to correspond with the necessary record.

Date, name, age, sex, temperament, name of tooth, pulp alive, pulp healthy, peridentium healthy, killed with arsenic, destroyed with instrument, arsenic caused pain hours, arsenic applied number of times, days in the tooth, pulp removed days after, root vessels alive, ditto removed, perfectly, medicated cavity with what, days of medication, plugged temporarily, date, plugged permanently, date, peridentitis slight, ditto severe, cured with what, abscess, date, default of patient, extracted. Most of these columns need be only one third of an inch wide.

My record for the treatment of alveolar abscess is ruled in a similar manner, with different headings.

Now I do not wish to influence you to adopt *my* practice; I have only stated it that you may compare it, if you have an opportunity, with that of others, and your own. But I desire you to distrust the statements of any one's practice, which is not made up from a regular *record*.

